# Introducing novel key genes and transcription factors associated with rectal cancer response to chemoradiation through co-expression network analysis

**DOI:** 10.1016/j.heliyon.2023.e18869

**Published:** 2023-08-02

**Authors:** Saeid Afshar, Tapak Leili, Payam Amini, Irina Dinu

**Affiliations:** a Cancer Research Center, Hamadan University of Medical Sciences, Hamadan, Iran; b Department of Biostatistics, School of Public Health and Modeling of Noncommunicable Diseases Research Center, Hamadan University of Medical Sciences, Hamadan, Iran; c School of Medicine, Keele University, Keele, Staffordshire, ST5 5BG, UK; d School of Public Health, University of Alberta, Edmonton, AB, Canada

**Keywords:** WGCNA, Colorectal neoplasm, Chemoradiotherapy, Transcription factors

## Abstract

Preoperative radiochemotherapy is a promising therapeutic method for locally advanced rectal cancer patients. However, the response of colorectal cancer (CRC) patients to preoperative radiotherapy varies widely. In this study, we aimed to identify novel biomarkers that could predict the response of colorectal tumors to treatment using a systems biology approach. We applied the Weighted Gene Co-Expression Network Analysis to construct co-expression networks and evaluated the correlation of these networks with radiation using the module-trait relationship. We then identified hub genes and related transcription factors in the selected co-expression module.

Our analysis of seven constructed modules revealed that one module, which contained 113 nodes and 6066 edges, had the strongest correlation with radiation effects on CRC (correlation = 0.85; p-value = 6e-7). By analyzing the selected module with the CytoHubba plugin, we identified four hub genes, including ZEB2, JAM2, NDN, and PPAP2A. We also identified seven important transcription factors, including KLF4, SUZ12, TCF4, NANOG, POU5F1, SOX2, and SMARCA4, which may play essential roles in regulating the four hub genes.

In summary, our findings suggest that ZEB2, JAM2, NDN, and PPAP2A, along with the seven transcription factors related to these hub genes, may be associated with the response of colorectal tumors to chemoradiotherapy.

## 1 Introduction

Colorectal cancer (CRC) is a prevalent and deadly neoplasm worldwide. In 2018, there were 1.8 million new cases of CRC reported globally and a number of 881,000 CRC-related deaths in people of all ages and both sexes were reported [1,2]. Preoperative radiochemotherapy is a favored therapeutic method for treating locally advanced rectal cancer (LARC) among the different treatment methods available for colorectal cancer (CRC) [[3], [4], [5]]. CRC patients respond variably to preoperative radiotherapy. Moreover, a large proportion of CRC patients have a poor prognosis and tumor recurrence. Treatment for patients with radioresistant tumors has minimum benefits but maximum toxic effects [[6], [7], [8], [9]]. Recent studies have indicated that several oncogenes or tumor suppressor genes have a crucial role in the response to chemoradiotherapy. However, the precise molecular mechanism underlying this response is unclear. Therefore, further evaluation of the molecular mechanism involved in the response to treatment is necessary [10,11].

The Gene Expression Omnibus (GEO) contains several gene expression datasets related to the effects of radiotherapy or chemoradiotherapy on colorectal tumor gene expression. In each study, the expression levels of several genes at the mRNA level were evaluated in both responders and non-responders among CRC patients. Some differentially expressed genes (DEG) were selected as novel discriminating genes [[12], [13], [14], [15], [16], [17], [18]]. Ghadimi et al. in their study, indicated that expression levels of 54 genes were significantly different between the two patients groups [12]. Watanabe et al. evaluated the effect of preoperative radiotherapy on gene expression in colorectal tumors. Their results indicated that the expression levels of 33 novel genes were statistically different between the two patients groups [13]. The results of Kim et al.’s study indicated that 95 top-ranked genes can predict responses to preoperative chemoradiotherapy in CRC patients [14]. Rimkus et al. evaluated the gene expressions related to the response to chemoradiotherapy in LARC patients by microarray in their study. Their results indicated that expression levels of 42 genes were significantly different between responders and non-responder in the patient population [16]. Nishioka et al. showed that the expression level of 17 genes can discriminate the response to preoperative chemoradiotherapy in rectal cancer patients [17]. Palma et al. evaluated the microarray gene expression and identified four genes as biomarkers of the response to preoperative chemoradiotherapy in locally advanced rectal cancer patients [18].

Weighted Gene Co-expression Network Analysis (WGCNA) is the weighted version of co-expression networks, in which relationship networks are constructed based on the pairwise or low-order conditional pairwise associations between gene expressions without any artificial cutoff. WGCNA has been proven to be a powerful tool in detecting co-expressed modules and hub genes in various disciplines [[19], [20], [21]]. WGCNA constructs groups of genes as a network using pairwise correlations between genes through the following steps: 1) defining the gene co-expression network based on the absolute value of the correlations between each paired genes; 2) defining the strength of the connection between genes using an adjacency matrix; 3) constructing a weighted network based on a soft thresholding parameter. These networks can be correlated to CRC. WGCNA utilizes a topological overlap measure as a proximity measure, so genes are clustered into network modules by combining the adjacency of every pair of genes and their connection strengths with other neighboring genes [22,23].

The module eigengene, which is the first principal component of the expression profiles, is calculated to summarize the genes inside a module. Then, the relationship between clinical outcomes and the modules is assessed using the correlation between module eigengenes and the outcomes. The intramodular hub genes are identified by calculating the correlation between genes and module eigengenes [22,24].

Co-expression networks, which are frequently used in biological studies, are constructed based on the correlation of DEGs [[25], [26], [27]]. WGCNA can be used to find co-expression modules in gene expression data and relate the identified modules to tumor traits, such as tumor stage, grade, metastasis [[28], [29], [30]]. However, despite several studies being conducted to understand the mechanisms of response to treatment, the accurate molecular mechanisms of the effects of chemoradiation on colorectal tumors have not been fully clarified. Thus, the main purpose of this study was to construct co-expression modules using gene expression data from CRC patients who underwent preoperative chemoradiotherapy using WGCNA, and subsequently identify the hub genes and related transcription factors.

## 2 Materials and methods

### 2.1 Microarray data

The gene expression profiling data of GSE15781 (platform: GPL2986, ABI Human Genome Survey Microarray Version 2) was originally obtained from the study by Snipstad et al. [3] and was retrieved from the GEO database (www.ncbi.nlm.nih.gov/geo/). The dataseries contains the 32,878 expression profile of 42 samples, including 13 non-irradiated colorectal tumors, 10 non-irradiated normal tissues, 9 irradiated colorectal tumors, and 10 irradiated normal tissues. Patients in the irradiated group receive a cumulative dose of 50 Gy of ionizing radiation and capecitabine during the treatment period.

### 2.2 Preprocessing and DEG analysis

After taking the log transform of the data, the limma package under Bioconductor in R software was applied to identify DEGs between non-irradiated colorectal tumors and non-irradiated normal tissues. The threshold for identifying DEGs was set at a p-value <0.05 adjusted based on the Benjamini-Hochberg procedure, and absolute log fold change (FC) > 1.

### 2.3 Gene co-expression network construction

The WGCNA package under R software was used for co-expression network construction. After an initial evaluation of non-irradiated tumor samples to identify outlier samples, the proper soft-threshold (power) was determined. Next, the dendrogram was constructed using hierarchical clustering with minimum module size = 30. In order to identify genes associated with the radiation response signaling pathway in colorectal tumors, we identified co-expression modules associated with irradiation. The correlation of each constructed module with irradiation was evaluated using the module-trait relationship. Subsequently, we evaluated the correlation of gene significance for treatment against membership for the module with the strongest module-trait relationship.

### 2.4 Hub genes and transcription factors identification

The constructed co-expression network of the selected module was exported and visualized by Cytoscape software version 3.6. The common hub genes determined by MNC, MCC, EPC, and degree algorithm through the CytoHubba plugin under Cytoscape software were selected for further evaluation.

### 2.5 The regulatory network of transcription factors and hub genes

The NetworkAnalyst 3.0 web-based tool (https://www.networkanalyst.ca/faces/home.xhtml) was applied for constructing the regulatory network of hub genes and related transcription factors. The constructed regulatory network was visualized with Cytoscape software. In order to identify transcription factors that have a greater interaction with hub genes, the transcription factors with a connection degree of more than 2 were selected.

### 2.6 Functional annotation analysis

The Kyoto Encyclopedia of Genes and Genomes (KEGG) pathway and Gene Ontology (GO) enrichment analysis were conducted for all DEGs using the DAVID database (https://david.ncifcrf.gov/). The KEGG pathway and GO analysis for all genes in the selected module were performed through the ClueGO Cytoscape plugin [[31], [32], [33]].

## 3 Results

### 3.1 Identification of DEGs

**Fig. 1:**
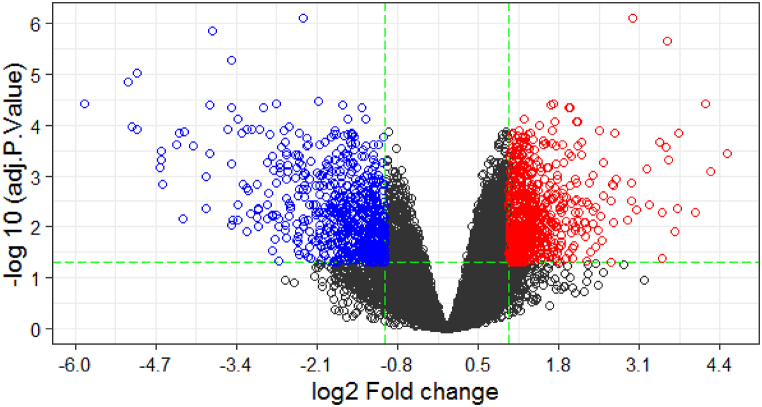
The Volcano plot for non-irradiated colorectal tumors compared with non-irradiated normal tissues. The blue circles are statistically significant downregulated genes, Red circles are statistically significant upregulated genes, and black circles are non-differentially expressed genes.

A total of 944 DEGs were identified in non-irradiated colorectal tumors compared with non-irradiated normal tissues. The Volcano plot was depicted by ggplot2 in R software by plotting the -log_10_ (adjusted *P*-value) versus log_2_(FC) (Fig. 1). The volcano plot is a commonly used visualization tool in transcritomics research that allows researchers to identify differentially expressed genes. In this plot, the x-axis represents the log2 fold change in gene expression between the two groups being compared (in this case, tumors vs. normal tissues), while the y-axis represents the statistical significance of the difference in expression levels (usually measured as -log10 p-value).

### 3.2 Weighted gene co-expression network analysis

**Fig. 2:**
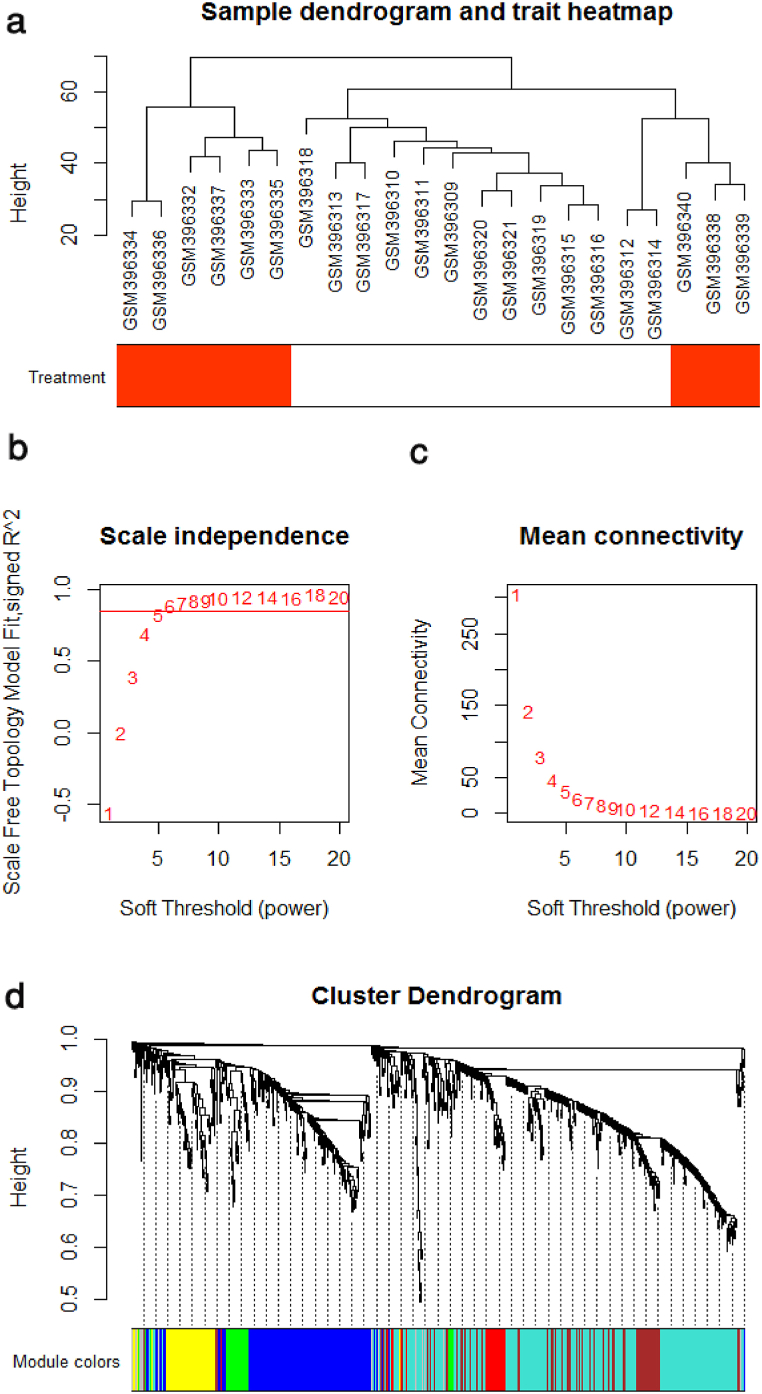
Clustering dendrogram and soft-thresholding (power) determination. (a) the expression data of DEGs between colorectal tumor samples and normal tissue samples were applied for clustering. (b) Analysis of the scale-free fit R^2^ for series of soft thresholds. (c) Mean connectivity analysis for series Powers. (d) Clustering dendrogram of all selected DEGs. Seven constructed co-expression modules were presented in different colors.

**Fig. 3:**
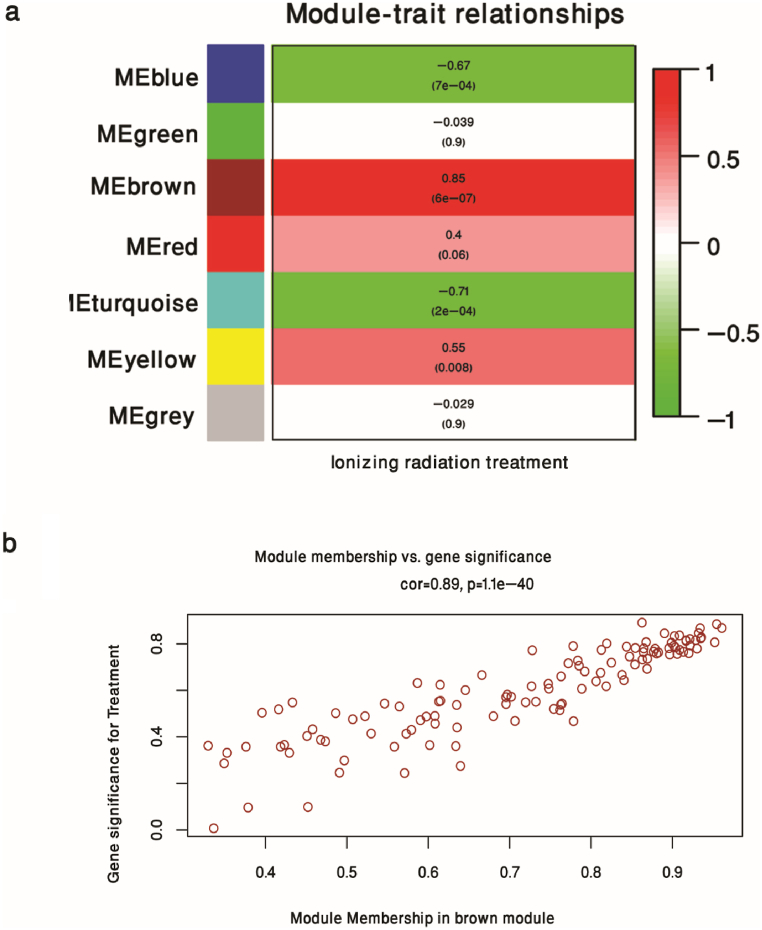
Relationships of co-expression modules with ionizing radiation treatment. (a) Module-trait relationship. The correlation coefficient of modules eigengenes and treatment were shown in each row and corresponding *P*-Value was shown in parentheses. (b) the scatter plot of module membership and gene significance for treatment in brown module.

The quality assessment checks by WGCNA indicated that all 22 colorectal tumor samples were included for subsequent analysis (Fig. 2a). The expression data of 944 DEGs of tumor samples were applied for co-expression network construction. In this study, the appropriate power was set to 6 by screening the power from 1 to 20, when the degree of independence was 0.85 (Fig. 2b–c). As seen in Fig. 2d, a total of seven co-expression modules were identified, with the size of the identified modules ranging from 25 to 397 genes. In order to find the modules which have a greater correlation with radiation effects on CRC, the module-trait relationship was evaluated (Fig. 3a). The brown module has the largest association with treatment (r = 0.85; *P*-Value = 6e-7). For further evaluation of the selected module, we plotted the gene significance for treatment against membership in the brown module. The correlation of gene significance vs. module membership was 0.89 (*P*-value = 1.1e-40) (Fig. 3b).

### 3.3 Functional annotation of DEGs and brown module

**Fig. 4:**
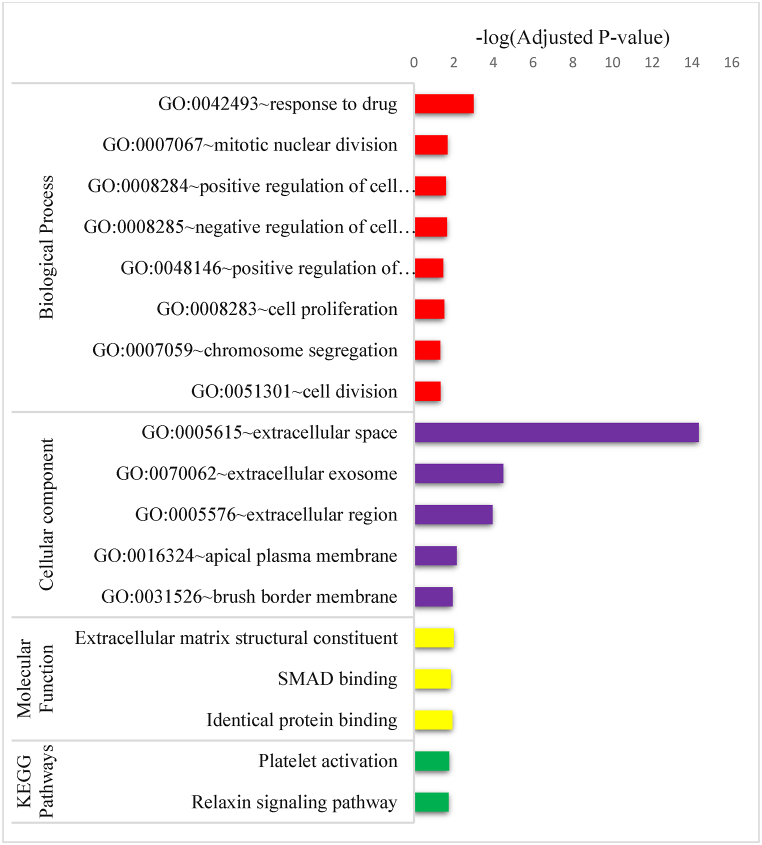
KEGG pathway and GO functional enrichment analysis of DEGs. The length of the bar represents the -log10 (*P*-value) of each pathway; GO biological process; GO molecular function; GO cellular Component, and KEGG pathways were shown on y-axis.

The GO functional enrichment analysis of DEGs showed that, in terms of biological processes, these genes are significantly enriched in response to drugs, mitotic nuclear division, negative regulation of cell proliferation, cell proliferation, chromosome segregation, positive regulation of cell proliferation, positive regulation of fibroblast proliferation, and cell division. In terms of cellular component GO terms, DEGs are enriched in the extracellular region, extracellular exosome, extracellular space, and apical plasma membrane. In terms of molecular function, the selected genes are enriched in hormone activity, CXCR chemokine receptor binding, and growth factor activity. Furthermore, KEGG pathway analysis indicated that DEGs are significantly enriched in mineral absorption and cell cycle (Fig. 4).

**Fig. 5:**
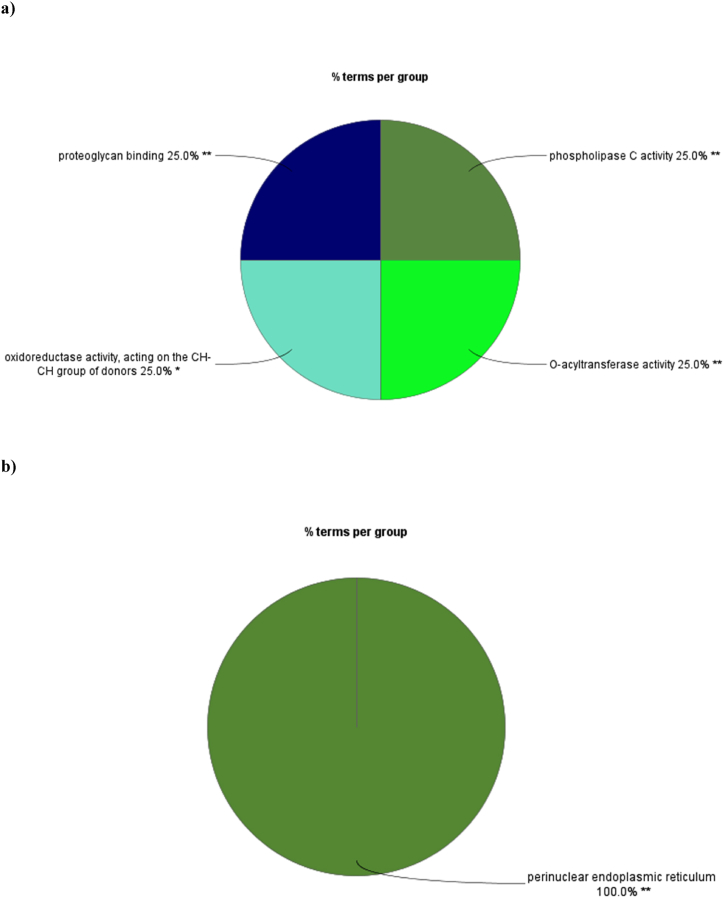

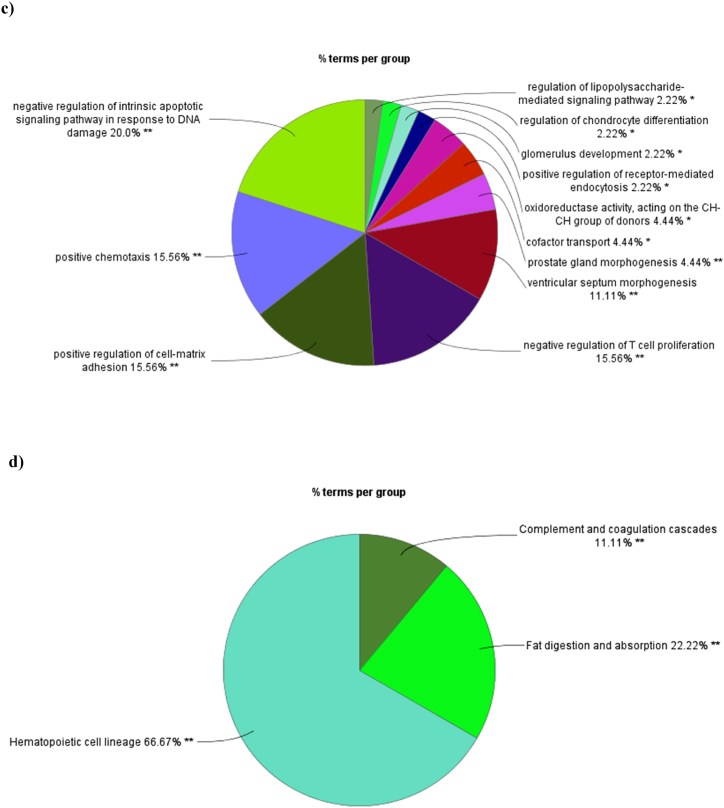
KEGG pathway and GO functional enrichment analysis of all genes in the brown module. The pie charts represent significantly overrepresented KEGG pathways and GO terms.

The GO term-related to molecular function, biological process, and cellular component, as well as the KEGG pathway pie charts for brown module genes, were constructed using the ClueGO plugin (Fig. 5a–d). The significant molecular function terms related to the brown module included phospholipase C activity, oxidoreductase activity on the CH–CH group of donors, O-acyltransferase activity, and proteoglycan binding. These molecular function terms, which occupy 20% of the pie chart, represent the main biological processes related to the brown module. The related KEGG signaling pathways for this module are Complement and coagulation cascades, Hematopoietic cell lineage, and fat digestion and absorption.

### 3.4 Hub genes identification

**Fig. 6:**
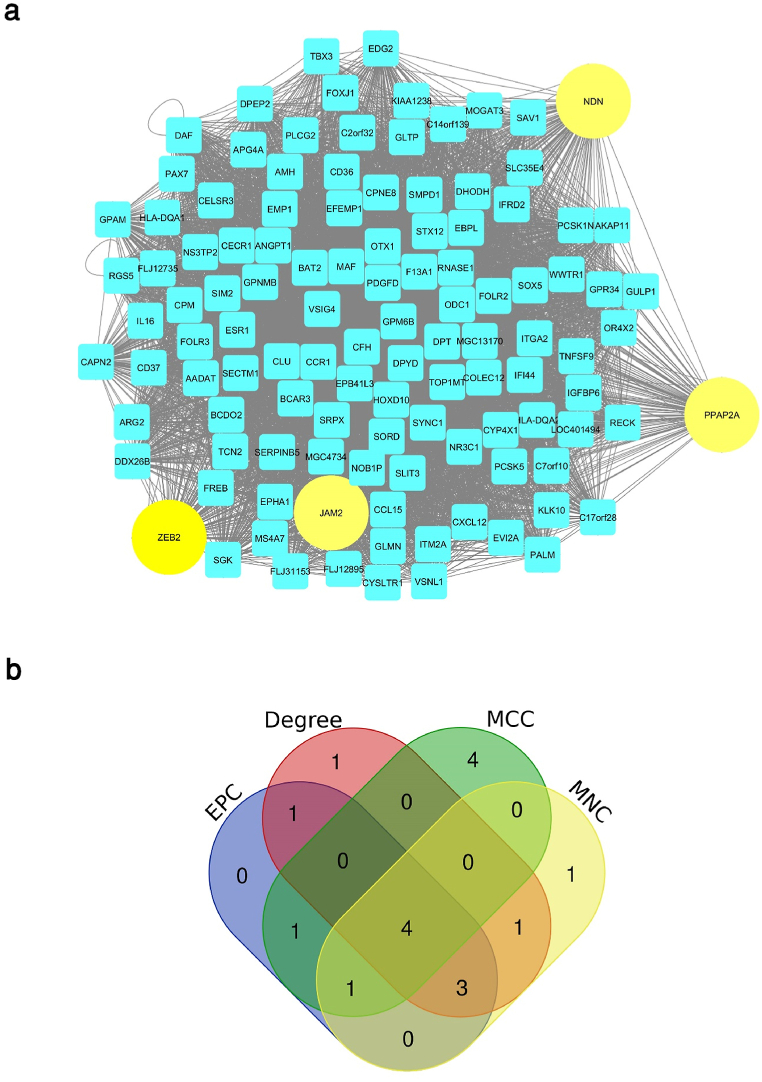
Constructed network and identified hub genes for the brown module. (a) the constructed co-expression network for the brown module contains 113 nodes and 6066 edges. Identified hub genes were shown in yellow circles. (b) Venn diagram was applied to show the common hub genes identified with Four calculation methods including MNC, MCC, degree, and EPC. Four hub genes in concurrent areas are ZEB2, JAM2, NDN, and PPAP2A.

The constructed network for the brown module, which includes 113 nodes and 6066 edges, was visualized and analyzed using Cytoscape 3.6 (Fig. 6a). Four calculation methods under the CytoHubba plugin, including MNC, MCC, degree, and EPC were used to identify hub gene (Table 1). After identifying the top 10 hub genes with each algorithm, the intersection of these methods was plotted as a Venn diagram (Fig. 6b). The common hub genes between four methods included Zinc Finger E-Box Binding Homeobox 2 (ZEB2), Junctional Adhesion Molecule 2 (JAM2), Necdin (NDN), and phosphatidic acid phosphatase type 2A (PPAP2A).

**Table 1:** Four hub genes identified with 4 calculation methods (MNC, MCC, degree, and EPC).

Gene symbol	MNC score	MCC score	EPC score	Degree
JAM2	40	356	43.885	41
NDN	30	208	41.887	30
PPAP2A	27	143	39.979	28
ZEB2	27	152	37.711	29

### 3.5 The regulatory network of transcription factors and hub genes

**Fig. 7:**
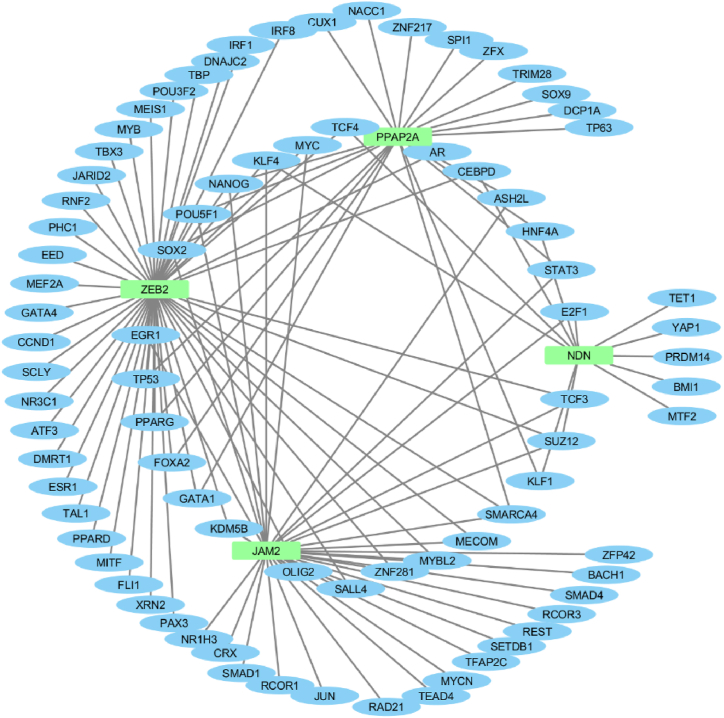
The regulatory network of hub genes and transcription factors. The hub genes were shown in green color and transcription factors were shown in blue color.

A regulatory network of the four selected genes and 82 related transcription factors was constructed by using NetworkAnalyst web-based tool. The constructed regulatory network contains 86 nodes and 116 edges (Fig. 7). The ZEB2, JAM2, PPAP2A, and NDN were regulated by 48, 33,23, and 12 transcription factors, respectively. As seen in Table 2, transcription factors including Krüppel-like Factor-4 (KLF4), suppressor of zest 12 (SUZ12), T cell factor 4 (TCF4), SRY-box 2 (SOX2), NANOG, SMARCA4, and POU domain class 5 transcription factor 1(POU5F1) have a connection degree of 3.

**Table 2:** The transcription factors of hub genes with connection degree ≥2.

Transcription factor	degree	Hub genes	Transcription factor	degree	Hub genes
KLF4	3	JAM2,NDN,ZEB2	FOXA2	2	ZEB2,PPAP2A
SUZ12	3	JAM2,NDN,ZEB2	GATA1	2	ZEB2,PPAP2A
TCF4	3	NDN,ZEB2,PPAP2A	MYC	2	JAM2,ZEB2
NANOG	3	JAM2,ZEB2.PPAP2A	TCF3	2	JAM2,ZEB2
POU5F1	3	JAM2,ZEB2.PPAP2A	EGR1	2	JAM2,ZEB2
SOX2	3	JAM2,ZEB2.PPAP2A	KDM5B	2	JAM2,ZEB2
SMARCA4	3	JAM2,ZEB2.PPAP2A	OLIG2	2	JAM2,ZEB2
CEBPD	2	NDN,ZEB2	SALL4	2	JAM2,ZEB2
E2F1	2	NDN,JAM2	ZNF281	2	JAM2,ZEB2
STAT3	2	JAM2,PPAP2A	MYBL2	2	JAM2,ZEB2
ASH2L	2	JAM2,PPAP2A	MECOM	2	JAM2,ZEB2
AR	2	ZEB2,PPAP2A	HNF4A	2	NDN,PPAP2A
TP53	2	ZEB2,PPAP2A	KLF1	2	NDN,PPAP2A
PPARG	2	ZEB2,PPAP2A			

## 4 Discussion

Generally, the prognosis of patients with radioresistant colorectal tumors is poor. Therefore, revealing the underlying molecular mechanism of response to radiation in colorectal tumors can aid in overcoming therapeutic obstacles and improving treatment strategies [15,34]. In this study, bioinformatics analysis was performed to identify essential genes and transcription factors involved in the response to radiation in colorectal tumors.

During the primary screening of GSE15781, a total of 944 DEG between tumor tissues and normal tissues were determined. GO enrichment analysis showed that these 944 genes were enriched in response to drug, negative regulation of cell proliferation, cell proliferation, chromosome segregation, positive regulation of cell proliferation, mitotic nuclear division, positive regulation of fibroblast proliferation, and cell division. Our results of GO enrichment analysis are consistent with those of other studies [[35], [36], [37]]. Similarly, our KEGG pathway enrichment analysis findings are consistent with existing literature on CRC, indicating that DEGs are enriched in cell cycle and mineral absorption pathways [38].

Our results indicated that among the co-expression modules for 944 DEGs using WGCNA, the brown module, including 113 genes, has the strongest correlation with treatment. GO enrichment analysis indicated that the major biological process related to DEGs is the negative regulation of intrinsic apoptotic signaling pathway in response to DNA damage. Similar studies have indicated that the cellular response to DNA damage stimulus term is similarly enriched for genes after ionizing radiation [39,40].

Evaluation of the brown module by network analysis indicated that ZEB2, JAM2, NDN, and PPAP2A are the hub genes with a potential role in the response to ionizing radiation in CRC. The gene ZEB2 has an essential role in proliferation, angiogenesis, metastasis, and epithelial-mesenchymal transition (EMT) of CRC, and has been previously found to be upregulated in CRC [41]. Results of recent studies have shown that ZEB1 and ZEB2 are associate with the response to radiotherapy and chemotherapy of several cancers, such as pancreatic carcinoma, head and neck squamous carcinoma, squamous carcinoma, breast cancer, non-small cell lung, and bladder carcinoma [42]. JAM2 belongs to the JAMs family and immunoglobulin subfamily, and it has an essential role in maintaining the integrity of cell-cell junctions and regulating angiogenesis and proliferation of tumor cells. Furthermore, the expression level of this gene is related to metastasis and prognosis of CRC patients [43]. NDN, as a novel target of P53, controls apoptosis and growth arrest of tumor cells. This gene associates with radioresistance of human fibroblast cells by affecting the signaling pathway of P53 in response to radiation-induced DNA damage [44]. In the study by Asai et al. the results indicated that NDN can regulate the sensitivity of hematopoietic stem cells to radiotherapy and chemotherapy by cell cycle-dependent and independent mechanisms [45]. PPAP2A (LPP1) degrades the lipid lysophosphatidate (LPA), which is mainly generated by the autotaxin (ATX) enzyme. LPA affects cellular function through the activation of LPAR1. LPAR1 activation by LPA leads to chemoresistance and radioresistance of tumor cells by upregulating nuclear factor erythroid 2-related factor 2 (Nrf2), multidrug resistance transporters, and anti-oxidant proteins [46,47].

The activation of transcription factors in response to ionizing radiation can influence the transcription of several genes that play a role in DNA damage response [48]. In this study, the most important transcription factors that may have an essential role in regulating four hub genes are KLF4, SUZ12, TCF4, NANOG, POU5F1, SOX2, and SMARCA4. KLF4, a zinc finger transcription factor, regulates the proliferation, migration, invasion, and apoptosis of colorectal tumor cells [49]. On the other hand, KLF4 as a radioprotective transcription factor in response to ionizing radiation has an anti-apoptotic activity [50]. SUZ12, as a main constituent of Polycomb repressive complex 2 (PRC2), is related to the invasion, metastasis, proliferation, and apoptosis inhibition of tumor cells [51]. Furthermore, this transcription factor has an essential role in the repair of DNA double-strand break (DSBs) and, therefore, in the response to ionizing radiation [52]. TCF4, a transcription factor of several Wnt signaling pathway genes, plays an important role in the response to chemoradiotherapy and prognosis of CRC patients [53]. In their study, Kendziorra et al. showed that the expression level of TCF4 was associated with the response to chemoradiotherapy in CRC by affecting cell cycle progression after irradiation [54]. Nanog, an essential regulator of self-renewal, has an important role in tumorigenesis. Moreover, the expression level of Nanog associates with chemoresistance and radioresistance of tumor cells [55]. Dehghan Harati et al. in their study indicated that Nanog has an essential role in controlling the radioresistance of breast cancer cells and ALDH activity, which is associated with DSB repair [56]. POU5F1 (OCT-4) regulates the pluripotency of embryonic stem cells. The expression level of POU5F1 is associated with the prognosis of CRC patients and with radioresistance of lung adenocarcinoma [57,58]. The protein encoded by POU5F1 has an essential role in regulating self-renewal, proliferation, DSB repair, and EMT of tumor cells. Hence, this gene, as a critical regulator of stemness, contributes to the response to ioinizing radiation [59]. SOX2 is one of the main regulators of pluripotency and self-renewal of stem cells. The expression level of SOX2 was associated with metastasis and prognosis of CRC patients [60]. SOX2 regulates the response to chemoradiotherapy and tumorigenesis of oral squamous cell carcinoma [61]. Results of Huang et al.’s study indicated that SOX2, which was associated with cancer stem cell phenotype, regulates the radioresistance of cervical cancer cells through the hedgehog signaling pathway and regulation of cell cycle progression, proliferation, and apoptosis of tumor cells [62]. SMARCA4 (BRG1), as a constituent of the chromatin-remodeling complex, plays a role in chromatin remodeling. The expression level of SMARCA4 was associated with prognosis of CRC patients [63]. SMARCA4 has an essential role in repair of DSB induced by ionizing radiation through the RAD51 and Ku loading [64]. Taken together, considering this evidence, these determined transcription factors may have a role in the response to ionizing radiation by regulating stemness properties, proliferation, apoptosis, and cell cycle progression. One important limitation of the present study was the limited dataset available for conducting WGCNA to analyze the response to treatment. As a result, the sample size of the dataset used in this study was relatively small, which underscores the need for conducting larger studies on the response to treatment in CRC.

In summary, the evaluation of the co-expression network constructed with WGCNA indicated that four key genes, including ZEB2, JAM2, NDN, and PPAP2A, are associated with the response to ionizing radiation. Moreover, the top seven important transcription factors related to the four hub genes were identified: KLF4, SUZ12, TCF4, NANOG, POU5F1, SOX2, and SMARCA4. These identified hub genes and transcription factors may have a critical role in the response to treatment of colorectal tumors. However, further studies are required to validate these findings.

## Ethical approval

This study was approved by the Ethics Committee of Hamadan University of Medical Sciences (approval code: IR.UMSHA.REC.1402.299). All methods were performed in accordance with the relevant guidelines and regulations.

## Author contribution statement

Saeid Afshar: Conceived and designed the experiments; Performed the experiments; Analyzed and interpreted the data; Contributed reagents, materials, analysis tools or data; Wrote the paper.

Leili Tapak: Conceived and designed the experiments; Analyzed and interpreted the data; Contributed reagents, materials, analysis tools or data; Wrote the paper.

Payam Amini, Irina Dinu: Analyzed and interpreted the data; Wrote the paper.

## Data availability statement

Data associated with this study has been deposited at https://www.ncbi.nlm.nih.gov/geo/query/acc.cgi?acc=GSE15781.

## Declaration of competing interest

The authors declare that they have no known competing financial interests or personal relationships that could have appeared to influence the work reported in this paper.
